# Human FDC express PrPc *in* *vivo* and *in* *vitro*

**DOI:** 10.1155/2001/45454

**Published:** 2001

**Authors:** Caroline Thielen, Nadine Antoine, France Mélot, Jean-Yves Cesbron, Ernst Heinen, Rikiya Tsunoda

**Affiliations:** 1 lnstitute of Human Histology University of Liège rue de Pitteurs 20 Bat. L3 Liège B-4020 Belgium; 2 Neurodegenerative Infectous Disease Dept. of Physiopathology Institut Pasteur of Lille France; 3 Dept. of Histology School of Medicine Fukushima Medical University I-Hikarigaoka Fukushima 960-1295 Japan

**Keywords:** Follicular Dendritic Cells (FDC), germinal centre, human tonsil, immunolabelling, prion protein (PrPc)

## Abstract

Prion diseases are fatal neurodegenerative disorders caused by accumulation of abnormal prion protein (protease-resistant prion, PrPres). PrPres accumulation is also detected in lymphoid organs after peripheral infection. Several studies suggest that follicular dendritic cells (FDC) could be the site of PrPres retention and amplification.

Here we show that human follicular dendritic cells can express normal cellular prion protein (PrPc) both *in* *situ* and *in* *vitro*. When tonsillar cryosections were treated with anti-PrP antibody, the label was found on some very delicate cell extensions inside the lymphoid follicles, especially in the germinal centres. These extensions react with DRC1 antibody, used frequently to label FDC. Other structures labelled with anti-PrP antibody were the keratinocytes.

To confirm the ability of FDC to synthesise PrPc, we isolated FDC by a non-enzymatic procedure and cultured them. By cytochemistry and flow cytometry it was clearly shown that FDC do produce PrPc.


*Developmental Immunology*, 2001, Vol. 8(3-4), pp. 259-266
Reprints available directly from the publisher
Photocopying permitted by license only

© 2001 OPA (Overseas Publishers Association)
N.V. Published by license under
the Harwood Academic Publishers imprint,
part of The Gordon and Breach Publishing Group,
a member of the Taylor and Francis Group

### Human FDC express PrPc *in vivo* and *in vitro*

CAROLINE THIELEN[a*], NADINE ANTOINE[a], FRANCE MÉLOT[a], JEAN-YVES CESBRON[b], ERNST HEINEN[a] and RIKIYA TSUNODA[c†]

*[a]Institute of Human Histology, University of Liège, Belgium, [b]Neurodegenerative Infectous Disease, Dept. of Physiopathology, Institut Pasteur of Lille, France and [c]Dept. of Histology, School of Medicine, Fukushima Medical University, Japan*

Prion diseases are fatal neurodegenerative disorders caused by accumulation of abnormal prion protein (protease-resistant prion, PrPres). PrPres accumulation is also detected in lymphoid organs after peripheral infection. Several studies suggest that follicular dendritic cells (FDC) could be the site of PrPres retention and amplification.

Here we show that human follicular dendritic cells can express normal cellular prion protein (PrPc) both *in situ* and *in vitro*. When tonsillar cryosections were treated with anti-PrP antibody, the label was found on some very delicate cell extensions inside the lymphoid follicles, especially in the germinal centres. These extensions react with DRC1 antibody, used frequently to label FDC. Other structures labelled with anti-PrP antibody were the keratinocytes.

To confirm the ability of FDC to synthesise PrPc, we isolated FDC by a non-enzymatic procedure and cultured them. By cytochemistry and flow cytometry it was clearly shown that FDC do produce PrPc.

*Keywords:* Follicular Dendritic Cells (FDC), germinal centre, human tonsil, immunolabelling, prion protein (PrPc)

### INTRODUCTION

Prion diseases are characterised by accumulation of infectious particles of protease-resistant prion (PrPres) (Prusiner et al., 1982). This pathological isoform results from modification of normal cellular protein called PrPc. This protein is widely distributed in the brain and other organs notably in the lymphoreticular system.

The symptoms of prion diseases always occur after accumulation of PrPres in the nervous system, leading to observable pathological lesions. After peripheral infection, however, PrPres is detected during the pre-clinical stages in the spleen and other lymphoid organs. Abundant PrPres was recently detected, before the appearance of the clinical signs of the disease, in the germinal centres of tonsils (Hill et al., 1997) and appendixes (Hilton et al., 1998) from patients affected with nvCJD.

The prion diseases are now widely studied in experimental models. Inoculation of PrPres into wild-type mice induces the different symptoms, whereas injection into PrP-deficient mice leads neither death nor to infectivity. This demonstrates the major role played by PrPc (Bueler et al., 1993).

Results obtained with immunodeficient mouse models show that lymphoid organs are involved in various steps of disease development after peripheral

* Name and address of first author: Caroline Thielen, Institute of Human Histology, University of Liège, rue de Pitteurs, 20 Bat. L3, B-4020 Liège, Belgium. Phone: ++32.4.366.51.78, Fax: ++32.4.366.51.73, Caroline.Thielen@ulg.ac.be
† Name and address of corresponding author: Rikiya Tsunoda, MD & PhD, Dpt of Histology, School of Medicine, Fukushima Medical University, I-Hikarigaoka, Fukushima 960–1295, Japan. Phone: 81–24–548–2111, Fax: 81–24–548–3836, @: rtsunoda@cc.fmu.ac.jp inoculation (Cesbron et al., 1998; Aucouturier et al., 1999). It is not clear, however, which immune system cells play a key role in pathogenicity. Spleen fractionation studies have shown that infectivity is associated with the stromal fraction and with lymphocytes (Clarke and Kimberlin, 1984; Raeber et al., 1999). The incubation period in peripherally infected mice is unaffected by whole body irradiation (Fraser and Farquhar, 1987). Thus, the prion replication depends on PrPc and notably on a radiation-resistant fraction of the cell population of lymphoid tissues. Follicular dendritic cells (FDC) belong to this radio-resistant fraction and are stromal cells (Kinet-Denoël et al., 1982). SCID mice lacking functional B and T cells and also functional FDC are resistant to peripheral infection by scrapie agent but develop the disease after a bone marrow reconstitution (Fraser et al., 1996). Reconstitution restores the presence of mature T and B cells and induces differentiation of functional follicular dendritic cells (Kapasi et al., 1993).

Immunohistochemical staining with anti-PrP antibodies reveals an extraordinary accumulation of PrPres in the lymphoid follicles, especially in the form of a delicate network (Kitamoto et al., 1991; McBride et al., 1992; Klein et al., 1998; Jeffrey et al., 1999). These results strongly suggest that the follicular dendritic cells might support scrapie agent accumulation and replication in the peripheral lymphoid organs.

Herein the aim was to determine whether human FDC can express PrPc. At this end, we have immunolabelled cryosections of human tonsils. Furthermore, since FDC are located solely in the germinal centres in intimate contact with B cells, we isolated them and analysed their capacity to react with anti-PrP antibodies just after purification and after several days of culture. We report here that human FDC do indeed synthesize PrPc.

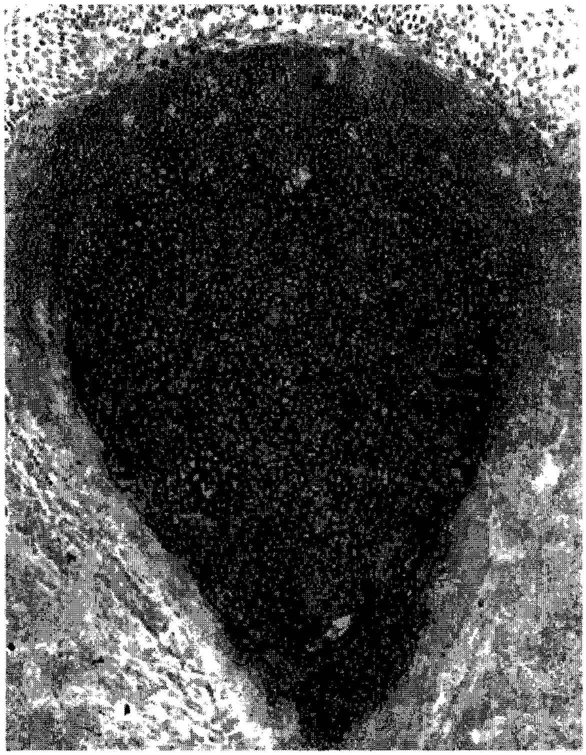

FIGURE 1 Immunolabelling of tonsillar follicular dendritic cells with MoAb: DRC-1 Counterstaining with haematoxylin (x 100)

### RESULTS

### FDC In Situ

On sections stained with MoAb:DRC1, a well-known marker of follicular dendritic cells, the network of cell extensions was intensely labelled (Fig. 1). After anti-PrP immunolabelling of cryosections with 3F4 antibody, the keratinocytes lining the crypts mainly at the level of basal cells were stained densely as it was previously reported by Pammer (Pammer et al., 1998), however, the germinal centres displayed a very low labelling. In the germinal centres, fine peroxidase reagent deposits were found along the intercellular spaces of the constituent cells (Fig. 2 A-B-C).

Control cryosections, reacted with normal mouse serum instead of anti-PrP antibody then with StrepAB Complex HRP and AEC didn't display any deposit in the germinal centres or along the crypts.

### Freshly Isolated FDC

FDC prepared by enzymatic digestion or mechanical dissociation showed engulfment of germinal centre

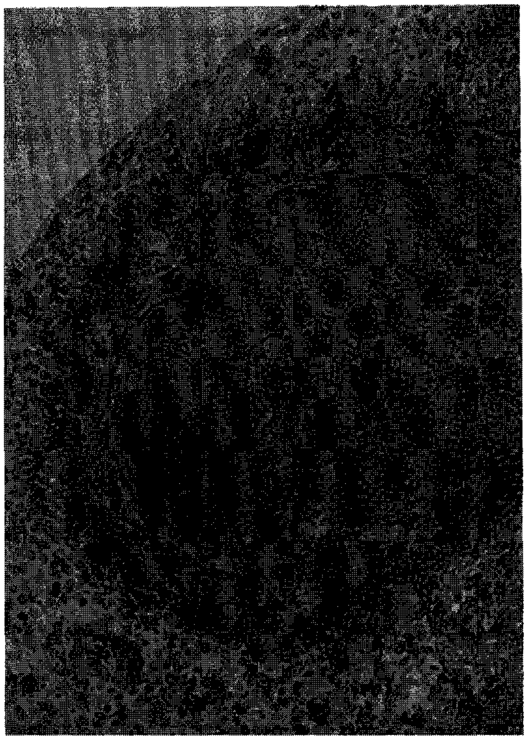

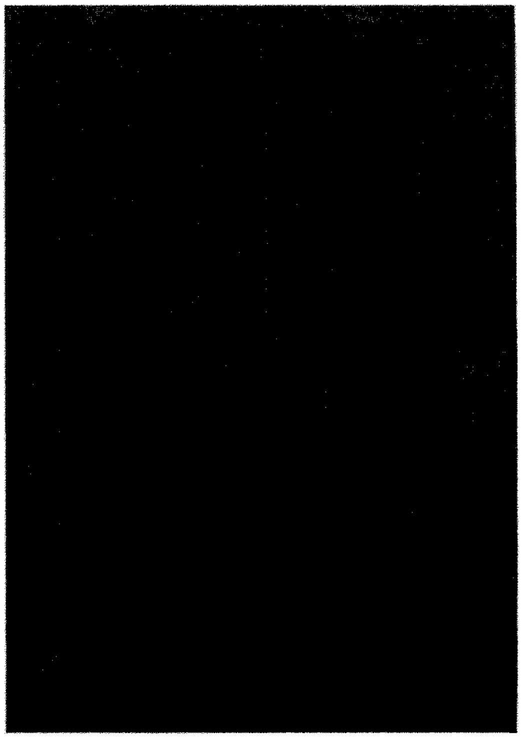

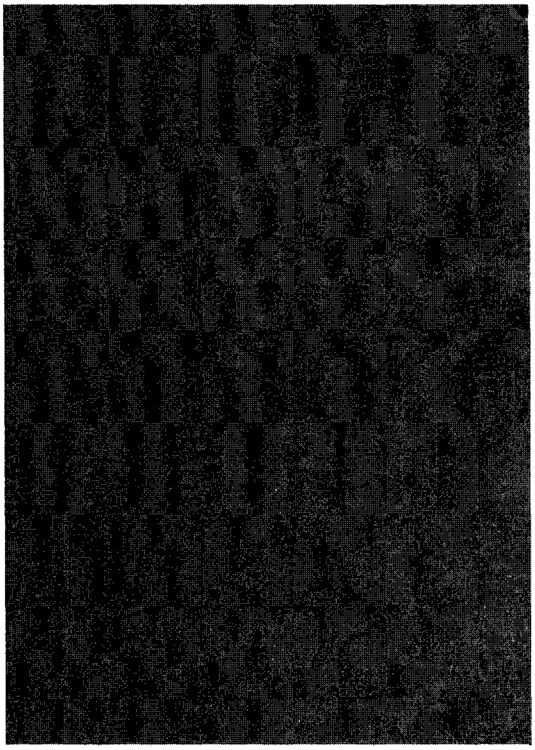

FIGURE 2 Immunolabelling of tonsillar cryosections with MoAb: 3F4. Counterstaining with haematoxylin. (A) Keratinocytes of the germinal layer in the crypts are positive (x 100). (B) Without counterstaining, a weak positive pattern is detected inside the germinal centre (x 650). (C) Besides the fine FDC extensions labelling, some stained cells (arrows) localised in the germinal centre present the morphology of tingible body macrophages (large binucleated cells)(x 650)

lymphoid cells by their cytoplasmic extensions. FDC always surrounded 3 to 20 lymphoid cells. These FDC-clusters could easily be distinguished from lymphocyte aggregates, vascular or epithelial fragments and tingible body macrophages. A strong positive reaction with DRC1 antibody was found on the cytoplasmic projections of these FDC, which were frequently bi-or multinucleated (Fig. 3).

PrPc expression was detected only on FDC-clusters isolated without enzymatic treatment. Weak staining with MoAbs:3F4, 3B5 and 12F10 was observed on the cytoplasmic projections of the FDC (Fig. 4 A-B).

The percentage and intensity of positive FDC-clusters depended to some degree on the monoclonal antibody used; 12F10 and 3F4 were recommended.

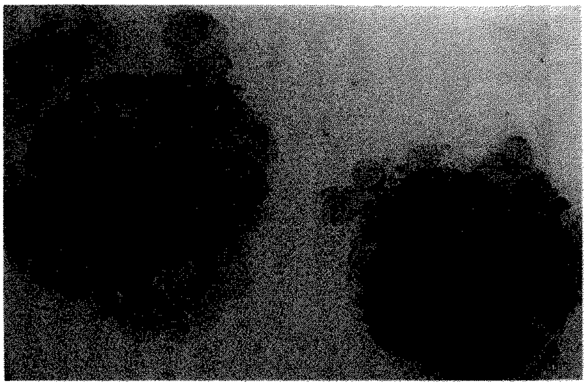

FIGURE 3 Immunostaining of freshly isolated FDC-cluster with MoAb: DRC1. Counterstaining with haematoxylin. DRC1 labelled cytoplasmic extensions surround numerous lymphocytes (x 400)

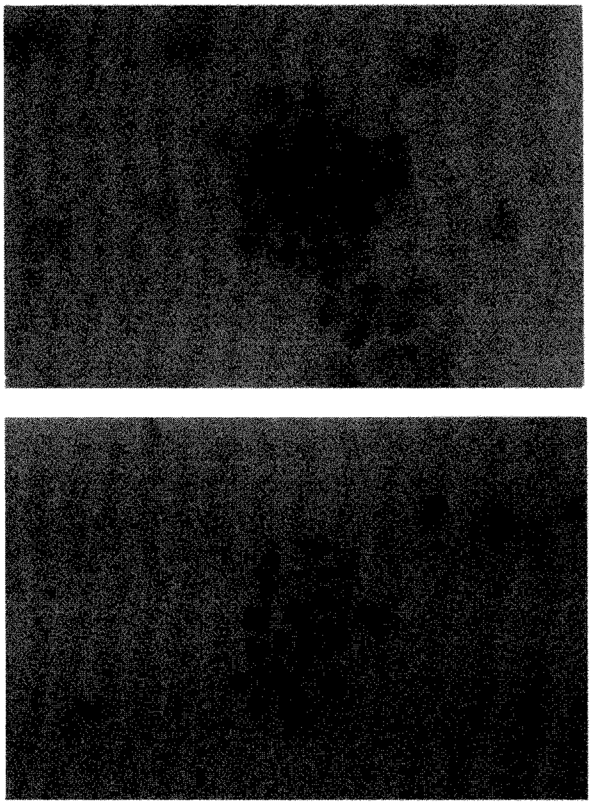

FIGURE 4 Immunostaining of FDC-cluster freshly isolated without enzyme with MoAbs: 3F4 (A) or 3B5 (B). Counterstaining with haematoxylin. Note the presence of a weak positivity on dendritic processes (x 400)

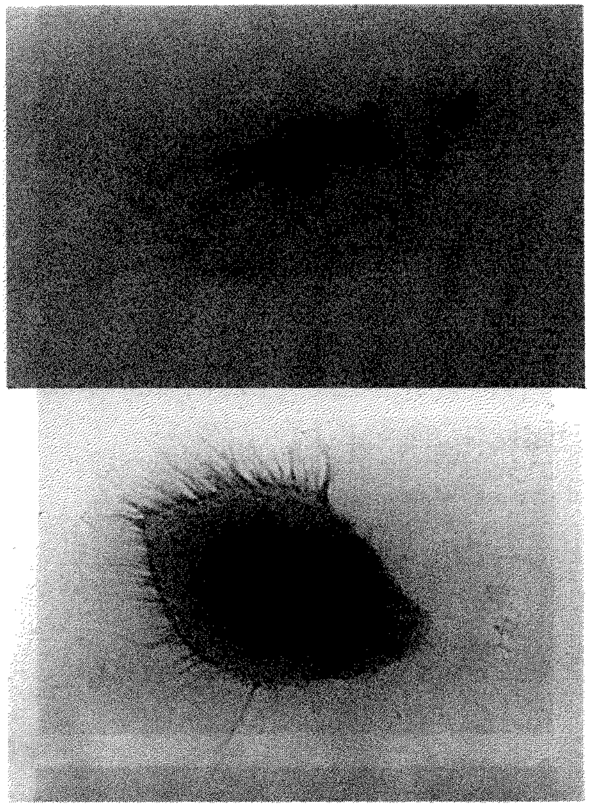

FIGURE 5 Immunostaining of 4 days-old cultured FDC with MoAbs: 12F10 (A) or 8G8 (B). Counterstaining with haematoxylin. During culture, FDC attach to the substrate and emit thin cytoplasmic extensions which are PrPc positive (x 650)

### Cultured FDC

During several hours of primary culture, the FDC extended thin cytoplasmic processes and adhered to the plastic surface. The engulfed lymphocytes eventually died by apoptosis. These FDC showed no proliferative activity and possessed one to ten nuclei.

After 4 days in culture, most of FDC reacted with 12F10 and 8G8 monoclonal antibodies in immunolabelling experiments (Fig. 5 A-B). Expression of PrPc was detected over the entire cell surface. Staining appeared more intense than when freshly prepared FDC-clusters were used.

PrPc expression was also analysed by cytometry on FDC cultured for 6 days. Since FDC *in vitro* adhere to the substrate, it was necessary to use trypsine to

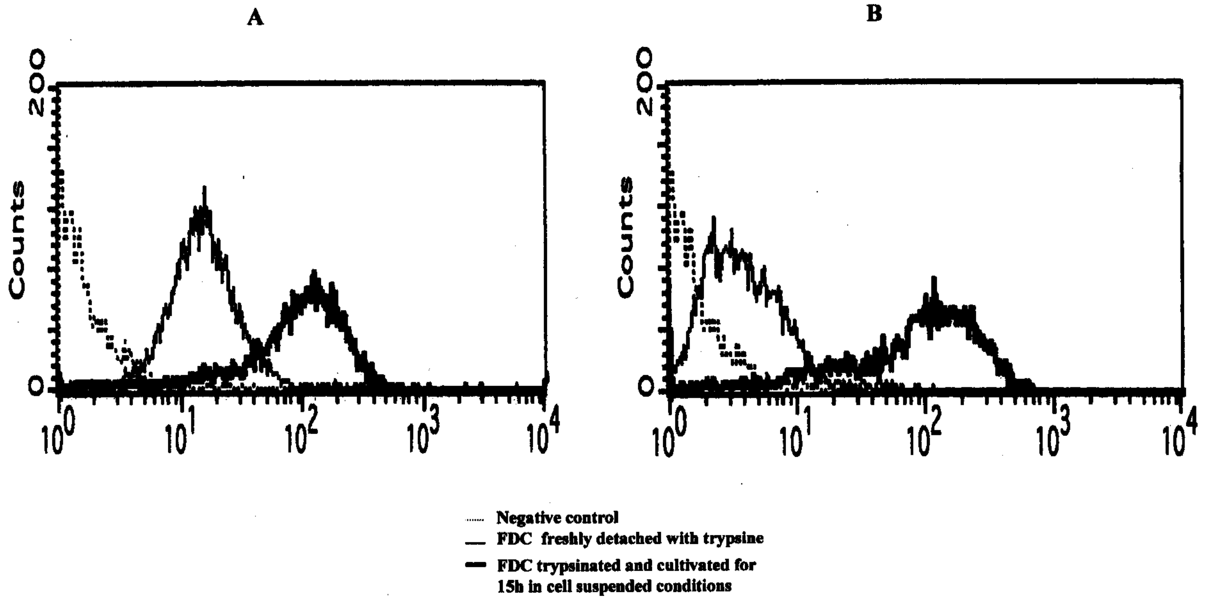

FIGURE 6 with MoAbs: 3B5 (A) and 848 (B). PrPc expression on 6 days-old cultured FDC analysed by cytometry detach them. The intensity of PrPc expression on freshly trypsinised FDC was low for both 3B5 and 8G8 monoclonal antibodies. To confirm that the FDC synthesise PrPc, we cultivated the trypsinised FDC in cell suspension conditions for an additional 15 h. This procedure allowed a re-expression of PrPc by FDC (Fig. 6 A-B). PrPc was detected with both antibodies and labelling was intense as with Jurkat cells (Fig. 7).

### DISCUSSION

*In vivo* findings of this and other laboratories (Kitamoto et al., 1991; McBride et al., 1992; Klein et al., 1998) indicate the presence of PrPc in low quantity along the cytoplasmic extensions of follicular dendritic cells. Since FDC make intimate contact with lymphoid cells and since lymphoid cells express PrPc (Cashman et al., 1999; Mabbott et al., 1997; Dodelet and Cashman, 1998), these findings do not constitute conclusive evidence that FDC synthesise PrPc.

In the present study, we thus chose to work on FDC isolated from human tonsils. We isolated the FDC in two different ways: by enzyme treatment and mechanical disruption. The former method has the disadvantage of removing PrPc from the surface of the FDC, as shown here and with Jurkat cells (data not shown). The latter method yielded FDC-clusters in which the FDC did appear to express PrPc on their surface. The problem with these results is again the presence of lymphocytes entrapped in the dendritic processes of the FDC, leaving open the possibility that the PrPc might actually be synthesized by the lymphocytes, then transferred to the FDC.

The question was thus: can FDC produce PrPc by themselves? To answer this question, we cultured these isolated FDC-clusters so as to eliminate all engulfed lymphocytes. In the culture system developed previously in this laboratory, FDC adhere to the culture substrate. The FDC stretch out and take a fibroblastic-like morphology. The lymphocytes eventually disappear by apoptosis so that only flat, firmly attached FDC remain in the culture system. On these attached FDC, we again detected PrPc by immunohistochemistry. Secondly, to analyse these cultured FDC by cytometry, we detached them with the aid of trypsine. Since this treatment removes the surface PrPc, as seen by cytometry and as reported by Caughey

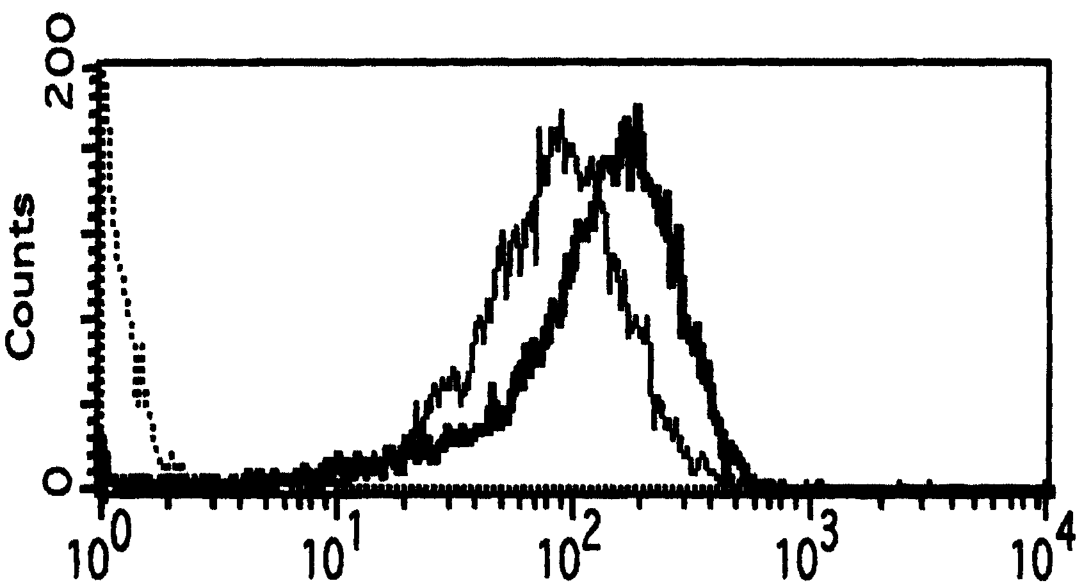

FIGURE 7 PrPc expression by Jurkat cells analysed by cytometry after MoAbs: 3B5 (line) or 8G8 (bold line) staining. Negative controls with a non-specific murine Ig-Per-Cys (dotted line)

(Caughey et al., 1988), we further cultured the detached FDC in cell suspension to give them time to synthesise PrPc and express it at their surface. The cultured FDC were PrPc-positive in absence of any lymphoid cells or stimulant. Thus, FDC appear to express PrPc independently of any stimulation or interaction with other cells. It is notheworthy that cultured lymphoid cells can also synthesise PrPc. Antoine et al. (submitted) working on cultured and freshly isolated tonsillar cells observed by cytometry higher surface expression of PrPc on the former than on the latter. Thus, *in vitro* expression of PrPc is independent of cell activation and may be related to stress or to loss of the *in vivo* microenvironment. In this work, furthermore, we observed different reactions according to the antibody used. These differences might reflect epitope accessibility related to protein folding or its glycosylation.

By long-term culturing of FDC, we have thus shown that these cells can express PrPc. In the future, this model will enable us to study PrPres replication and infectivity in FDC *in vitro*.

### MATERIALS AND METHODS

### Isolation and Purification of FDC-clusters

Palatine tonsils were surgically removed from children aged 3 to 10. FDC-clusters were isolated by two different procedures, enzymatically for culturing and non-enzymatically for immunostaining on cytospins.

Enzymatic digestion method was described elsewhere (Tsunoda et al., 1990); the tonsils were cut in slices (1 mm thick) and digested for 20 min at 37°C with an enzyme cocktail containing 0.1 % collagenase (Type A, Boehringer-Mannheim), 0.05 % dispase II (Grade II, Boehringer-Mannheim), 0.06 % DNase I (Grade II, Boehringer-Mannheim) in PBS with 0.4 % Bovine Serum Albumin (BSA). The freed-cells fraction was stored in cold PBS with 0.4 % BSA. The remaining tissues were digested again with fresh enzyme solution.

For non-enzymatic dissociation, we chose gross dissection and mechanical disruption of lymphoid follicles. After the enucleating the follicles under anatomical microscope, we squeezed them between two glass slides to obtain a cell suspension composed mainly of germinal-centre cells. The cell preparation was filtrated on gauze to eliminate collagen bundles and blood vessels. The whole procedure was carried out in PBS + 0.4 % BSA at 4°C.

After both cell dissociation procedures, the preparations were enriched in FDC-clusters by repetitive 1G sedimentation through Fetal Calf Serum (FCS) (Wekerle et al., 1980). FDC-clusters were finally enriched at approximately 96% (v/v ratio).

### FDC Culture

The final FDC-cluster rich fraction was incubated in a plastic culture dish for 60 min with RPMI 1640 with 10 % FCS at 37°C, 5 % $CO_2$ to remove contaminating macrophages. Next, non-adhering cells were transferred to new wells for a 6-h incubation in fresh medium: RPMI 1640, 2 mM L-glutamine, 50 μM 2-mercaptoethanol, 100 U/ml penicillin, 100 μg/ml streptomycin, 5 % FCS. During this incubation, FDC adhered weakly to the substrate. Non-adhering population was carefully discarded. The culture medium was replaced once daily.

For immunohistochemistry, some FDC-clusters were cultured on polylysine-coated glass coverslips in 6-well culture plates.

### Immunohistochemistry

Cryosections (8–10 μm) of tonsils and cytospins of FDC-clusters (non-enzymatically isolated) were fixed in acetone for 10 min at 4°C. On the other hand, FDC cultured on polylysine-coated glass coverslips for 4 days were fixed in paraformaldehyde 2 % for 10 min at 4°C.

Cryosections and cytospecimens were treated with a non-specific rabbit serum for 15 min, then allowed to react for 1 h at room temperature with MoAbs: DRC1 (Dako, Denmark), 12F10, 3B5, 8G8 or 3F4 antibody at optimal dilution (1/100, 1/100, 1/100, 1/100 and 1/200 respectively). The preparations were incubated with biotinylated rabbit anti-mouse immunoglobulin (αIg, 1/200) for 1 h, followed by StrepAB Complex HRP (Zymed). Peroxidase activity was revealed with 9-ethyl-3-aminocarbazol (AEC) and $H_2O_2$ as substrates (Zymed). For the cryosections, we used an amplification system (Envison kit, Dako, Denmak) in place of the secondary antibody. All antibody dilutions and wash steps were performed in phosphate buffered saline, pH 7.2 (supplemented with 0.5 % Tween for MoAb 3F4 dilution only).

Negative controls consisted of preparations in which the specific antibody was replaced with non-specific mouse serum and once from which the primary antibody was omitted.

### Flow-cytometric Analysis

After 6 days of culture, the FDC were checked for the ability to effect B lymphocytes emperipolesis. This is a reliable test for identifying FDC *in vitro* (Tsunoda et al., 1992). Freshly prepared tonsillar B lymphocytes were added to the monolayered FDC *in vitro*. Cultured FDC can entrap lymphocytes beneath their cytoplasm extensions (pseudoemperipolesis). Other FDC that had been cultured for 6 days were harvested by treatment with 0.25 % trypsin and 0.04 % ethylenediamine tetra-acetic acid in PBS and washed. Some detached FDC were stored in FCS on ice until immunostaining. Others were cultured for 15 h at 37°C as cell suspensions in RPMI containing 10 % FCS and HEPES. This was done with the Techne® system (Duxford Cambridge, UK) avoiding attachment to the substrate. Thereafter, the cells were collected and washed. Both types of FDC preparation were incubated with biotinylated monoclonal antibody (8G8 or 3B5), followed by Streptavidin-Per-Cys (Dako, Denmark). Jurkat cells (human T-cell lymphoma-cell line) constituted the positive control for PrPc. In the negative control, we used non-specific murine IgG-Per-Cys (Dako, Denmark).

The fluorescence intensity was analyzed by means of the Cell-Quest system with a FACScan flow cytometer (Becton-Dickinson, USA).

### *Acknowledgements*

We acknowledge the technical assistance of S. Collin, B. Coumans and M. Jackers, We thank Professor A. Aguzzy for the 3F4 antibody. The 12F10, 8G8 and 3B5 antibodies were provided by CEA Saclay, France. C. Thielen is the recipient of a grant from F.R.I.A. (Fonds pour la Recherche Industrielle et Agricole) and R. Tsunoda was supported by a grant in aid from the Ministry of Education, Science and culture of Japan.

### *References*

Aucouturier P., Frangione B. and Wisniewski T. (1999). Prion diseases and the immune system. Ann. Med. Interne **150**:75–78.

Bueler H., Aguzzi A., Sailer A., Greiner R.A., Autenried P., Aguet M. and Weissmann C. (1993). Mice devoid of PrP are resistant to scrapie. Cell **73**:1339–1347.

Cashman N.R., Loertscher R., Nalbantoglu J., Shaw I., Kascsak R.J., Bolton D.C. and Bendheim P.E. (1990). Cellular isoform of the scrapie agent protein participates in lymphocyte activation. Cell **61**:185–192.

Caughey B., Race R.E., Vogel M., Buchmeier M.J. and Chesebro B. (1988). In vitro expression in eukariotic cells of prion protein gene cloned from scrapie-infected mouse brain. Proc. Natl. Acad. Sci. U.S.A. **85**:4657–4661.

Cesbron J.Y., Lemaire C., Delhem N., Schulze T., Blanquet F. (1998). Rôle du système immunitaire dans les maladies à prions. Med. et Science **14**:1204–1210.

Clarke M.C. and Kimberlin' R.H. (1984). Pathogenesis of mouse scrapie: distribution of agent in the pulp and stroma of infected spleens. Vet. Microbiol. **9**:215–225.

Dodelet V.C. and Cashman N.R. (1998). Prion protein expression in human leukocyte differentiation. Blood **91**:1556–1561.

Fraser H., Brown K.L., Stewart K., McConnell I., McBride P. and Williams A. (1996). Replication of scrapie in spleens of SCID mice follows reconstitution with wild-type mouse bone marrow. J. Gen. Virol. **77**:1935–1940.

Fraser H. and Farquhar C.F. (1987). Ionising radiation has no influence on scrapie incubation period in mice. Vet. Microbiol. **13**:211–223.

Hill A.F., Zeidler M., Ironside J. and Collinge J. (1997). Diagnosis of new variant Creutzfeldt-Jakob disease by tonsil biopsy [see comments]. Lancet **349**:99–100.

Hilton D.A., Fathers E., Edwards P., Ironside J.W. and Zajicek J. (1998). Prion immunoreactivity in appendix before clinical onset of variant Creutzfeldt-Jakob disease [letter]. Lancet **352**:703–704.

Jeffrey M., McGovern G., Brown K.L., Goodsir C.M., Bruce M. and Brown J. (1999). Ultrastructural localisation of PrP in murine scrapie infected spleen. abstract Germinal Centre Conference.

Kapasi Z.F., Burton G.F., Schultz L.D., Tew J.G. and Szakal A.K. (1993). Induction of functional follicular dendritic cells development in severe combined immunodeficiency mice: Influence of B and T cells. J. Immunol. **150**:2648–2658.

Kinet-Denoël C., Heinen E., Radoux D. and Simar L.J. (1982). Follicular dendritic cells in lymph nodes after X-irradiation. Int. J. Radiat. Biol. **42**:121–130.

Kitamoto T., Muramoto T., Mohri S., Doh-ura K. and Tateishi J. (1991). Abnormal isoform of prion protein accumulates in follicular dendritic cells in mice with Creutzfeldt-Jakob disease. J. Virol. **65**:6292–6295.

Klein M.A., Frigg R., Raeber A.J., Flechsig E., Hegyi I., Zinkernagel R.M., Weissmann C. and Aguzzi A. (1998). PrP expression in B lymphocytes is not required for prion neuroinvasion [see comments]. Nat. Med. **4**:1429–1433.

Mabbott N.A., Brown K.L., Manson J. and Bruce M.E. (1997). T-lymphocyte activation and the cellular form of the prion protein. Immunology **92**:161–165.

McBride P.A., Eikelenboom P., Kraal G., Fraser H. and Bruce M.E. (1992). PrP protein is associated with follicular dendritic cells of spleens and lymph nodes in uninfected and scrapie-infected mice. J. Pathol. **168**:413–418.

Pammer J., Weninger W. and Tschachler E. (1998). Human keratinocytes express cellular prion-related protein in vitro and during inflammatory skin diseases. Am. J. Pathol. **153**:1353–1358.

Prusiner S.B., Bolton D.C., Groth D.F., Bowman K.A., Cochran S.P. and McKinley M.P. (1982). Further purification and characterization of scrapie prions. Biochemistry **21**:6942–6950.

Raeber A., Klein M.A., Frigg R., Flechsig E., Aguzzi A. and Weissmann C. (1999). PrP-dependent association of prions with splenic but no circulating lymphocytes of scrapie-infected mice. The EMBO Journal **18**:2702–2708.

Tsunoda R., Nakayama M., Onozaki K., Heinen E., Cormann N., Kinet-Denoël C. and Kojima M. (1990). Isolation and long-term cultivation of human tonsil follicular dendritic cells. Virchows Arch B Cell Pathol **59**:95–105.

Tsunoda R., Nakayama M., Heinen E., Miyake K., Suzuki K., Sugai N., Kojima M. (1992) Emperipolesis of lymphoid cells by human follicular dendritic cells in vitro. Virchows Arch B Cell Pathol. 62: 69-78.

Wekerle H., Ketelsen U. and Ernst M. (1980). Thymic nurse cells. Lymphoepithelial cell complex in murine thymuses: morphological and serological characterization. J. Exp Med **151**:925–944.

